# Complete Closed Genome Sequence of the Inulin-Utilizing Lactiplantibacillus plantarum Strain Lp900, Obtained Using a Hybrid Nanopore and Illumina Assembly

**DOI:** 10.1128/MRA.00185-21

**Published:** 2021-04-29

**Authors:** Jori Fuhren, Reindert Nijland, Michiel Wels, Jos Boekhorst, Michiel Kleerebezem

**Affiliations:** a Host Microbe Interactomics Group, Wageningen University and Research, Wageningen, The Netherlands; b Marine Animal Ecology Group, Wageningen University and Research, Wageningen, The Netherlands; c NIZO food research B.V., Ede, The Netherlands; University of Arizona

## Abstract

Lactiplantibacillus plantarum is a genetically and phenotypically diverse lactic acid bacterium species. We announce the hybrid *de novo* assembly of Oxford Nanopore Technologies and Illumina DNA sequence reads, producing a closed circular chromosome of 3,206,992 bp and six plasmids of the inulin-utilizing L. plantarum strain Lp900.

## Abstract (precis)

Lactiplantibacillus plantarum is a genetically and phenotypically diverse species of lactic acid bacteria. We announce the hybrid *de novo* assembly of Oxford Nanopore Technologies and Illumina DNA sequence reads, producing a closed circular chromosome of 3,206,992 bp and six plasmids of the inulin-utilizing L. plantarum strain Lp900.

## ANNOUNCEMENT

Lactiplantibacillus plantarum is one of the most versatile species of lactic acid bacteria (LAB) found in fermented and unfermented foods and the human gastrointestinal tract, displaying remarkable phenotypic and genomic diversity (1–3). L. plantarum is a dominant species in fermentation processes (4–6), and strain-specific potential beneficial effects on health have been described before (7, 8). The first L. plantarum strain to be sequenced was the human saliva isolate WCFS1 (9); recently, a multitude of strains have been sequenced, allowing comparative genomic analyses and providing more insight into the genomic diversity of L. plantarum (2). L. plantarum strain Lp900 was isolated in Nigeria from ogi, a fermented cereal pudding produced from red sorghum grains (10), and it has shown the capacity to utilize inulin and fructo-oligosaccharides (11). It harbors several plasmids, one of which codes for an inducible operon consisting of a transcriptional regulator, a canonical phosphotransferase system (PTS), and an extracellular β-fructosidase (12).

L. plantarum strain Lp900 was obtained from the PROBI A/B culture collection, and minimal cultivation steps in inulin-supplemented laboratory medium were included prior to the isolation of total DNA for sequencing, as well as storage of the strain at −80°C in medium supplemented with 15% glycerol. Prior to DNA isolation, strain Lp900 was cultured in de Man, Rogosa, and Sharpe (MRS) broth overnight at 37°C and passaged in fresh medium, and cells were harvested during exponential growth, flash frozen, reheated at 65°C for 10 min, resuspended in THMS (25% sucrose, 300 mM Tris-HCl [pH 8.0]) containing 20 mg/ml lysozyme and 300 U mutanolysin, and incubated for 1 h at 37°C. Subsequently 1× TAE was added (final concentrations of 30 mM Tris, 15 mM sodium acetate, and 10 mM EDTA), and the sample was treated with SDS (final concentration of 1% [wt/vol]) at 65°C for 20 min. Afterwards, samples were incubated with RNase (0.1 mg/ml) and proteinase K (0.35 mg/ml) for 1 h and 30 min, respectively, at 37°C. Genomic DNA was extracted with phenol-chloroform, precipitated with absolute ethanol and sodium acetate (final concentration of 0.3 M), and dissolved overnight in sterile water (13).

Illumina DNA sequencing libraries were prepared with the Nextera XT library preparation kit (Illumina, USA) and sequenced using 125-bp paired-end libraries on an Illumina HiSeq 2500 system (Illumina) with a total number of 3,677,745 reads, with target coverage of ∼138×. Illumina sequencing was performed at BaseClear (Leiden, The Netherlands). Nanopore libraries were prepared using the SQK-LSK109 1D genomic DNA ligation kit (Oxford Nanopore Technologies, UK) with 1 μg of genomic DNA as the input and sequenced on a MinION Mk1 instrument using an R9.4.1 flow cell (Oxford Nanopore Technologies), which delivered >1 Gb of raw data consisting of 128,396 1D reads with a mean length of 8,057 kb. Base calling was performed on the MinIT device running Guppy v2.0.10. Raw data were filtered using Filtlong v0.2.0 (https://github.com/rrwick/Filtlong) down to 300 MB of data with predicted genome coverage of ∼90×. Extracted FASTA files from Illumina and Nanopore sequencing data were integrated in a hybrid genome assembly using Unicycler v0.4.8-beta (14), producing a complete circular chromosome (3,206,992 bp) and six plasmids varying between 5 and 51 kb, with a GC content of 44.37%. The complete L. plantarum genome was annotated with Prokka v1.13.3 (15).

### Data availability.

The complete genome sequence and plasmids of L. plantarum Lp900 were deposited in GenBank under BioProject number PRJNA630372 and BioSample number SAMN14833145. Raw sequences of both Illumina and Nanopore reads were submitted to the NCBI SRA under BioProject number PRJNA630372.
